# Effect of three different cultivars of *Lepidium meyenii *(Maca) on learning and depression in ovariectomized mice

**DOI:** 10.1186/1472-6882-6-23

**Published:** 2006-06-23

**Authors:** Julio Rubio, Maria Caldas, Sonia Dávila, Manuel Gasco, Gustavo F Gonzales

**Affiliations:** 1 Department of Biological and Physiological Sciences, Faculty of Sciences and Philosophy and Instituto de Investigaciones de la Altura, Universidad Peruana Cayetano Heredia. P.O. Box 1843, Lima, Peru

## Abstract

**Background:**

*Lepidium meyenii *Walp. (Brassicaceae), known as Maca, is a Peruvian hypocotyl growing exclusively between 4000 and 4500 m altitude in the central Peruvian Andes, particularly in Junin plateau and is used traditionally to enhance fertility. Maca is a cultivated plant and different cultivars are described according to the color of the hypocotyls.

**Methods:**

The study aimed to elucidate the effect of Yellow, Red and Black Maca on cognitive function and depression in ovariectomized (OVX) mice. In all experiments OVX mice were treated during 21 days and divided in four groups: control group, Yellow Maca, Red Maca and Black Maca. Latent learning was assessed using the water finding task and the antidepressant activity of the three varieties of Maca was evaluated using the forced swimming test. Animals were sacrificed at the end of each treatment and the uterus were excised and weighed.

**Results:**

Black Maca was the variety that showed the best response in the water finding task, particularly in the trained mice. The three varieties were effective to reduce finding latency in non trained and trained mice (P < 0.05). In the force swimming test, all varieties assessed reduced the time of immobility and increased uterine weight in OVX mice.

**Conclusion:**

Black Maca appeared to have more beneficial effects on latent learning in OVX mice; meanwhile, all varieties of Maca showed antidepressant activity.

## Background

*Lepidium meyenii *Walp. (Brassicaceae), known as Maca, is a Peruvian hypocotyl growing exclusively between 4000 and 4500 m altitude in the central Peruvian Andes, particularly in Junin plateau and is used traditionally to enhance fertility in men and women. In fact, Father Bernabe Cobo, a chronicler of the Spanish conquest of Peru, referred to the fertility-enhancing property of Maca during the first half of the seventeenth century [1].

Previous studies showed that hypocotyls of Yellow *Lepidium meyenii *(Yellow Maca) improved sperm production in rats [2-5], mice [6] and humans [7]. Also, Yellow Maca enhanced female fertility increasing litter size [8]. Moreover, Maca improved sexual performance parameters in mice [9] and rats [9-12].

Maca is a cultivated plant and different cultivars are described according to the color of its hypocotyls. In the Department of Junin (Carhuamayo), 13 varieties of Maca ranging from White to Black have been described. The most frequent was the Yellow color (47.8%), the most preferred commercially [12].

Different biological properties have been observed among different varieties of Maca. In fact, previous studies showed that Black Maca presented the greatest effect on spermatogenesis when compared with Yellow and Red Maca [13]. In addition, Red Maca, but neither Yellow nor Black Maca, significantly reduced prostate size in rats [14].

Although Maca has been traditionally described to be favorable for fertility [1], other properties have also been scientifically reported. For instance, men taking gelatinized Maca show lower scores for the Hamilton Rating Scale for Depression after four weeks of treatment [15,16]. Moreover, it was demonstrated the anti-stress activity of a methanolic extract of Maca in rats [17]. However, nothing is known about the differential effect of varieties of Maca.

The present study aimed to elucidate the effect of Yellow, Red and Black Maca on learning and depression in ovariectomized (OVX) mice.

## Methods

### Animals

Three-month-old female mice from the Swiss strain obtained from the animal house of the Universidad Peruana Cayetano Heredia were used for the study. Mice were housed 5 per cage and maintained at ambient temperature (22°C) with a 12:12 h light/dark cycle in the animal house at the Universidad Peruana Cayetano Heredia. Mice were provided with Purina laboratory chow and tap water *ad libitum*.

### Ovariectomy

Mice at the age of three months were anesthetized by intraperitoneal injection of a mixture of 40 mg/Kg of ketamine and 10 mg/kg of xylazine and ovariectomized using a dorsolateral approach. Animals were included in the experiments three months after they were ovariectomized.

### Preparation of aqueous extract of *Lepidium meyenii *(Maca)

The dried hypocotyls of *Lepidium meyenii *(Brassicaceae) were obtained from Carhuamayo, Junin at 4000 m altitude. Irma Fernandez, who is a Botanist of the Department of Pharmaceutical Sciences, Universidad Peruana Cayetano Heredia, authenticated the identity of the plant. The voucher number IFV 1885 was deposited at the Department. Biological activity of the plant is located in the hypocotyls that are consumed by natives after being naturally dried. Traditionally, the dried hypocotyls of Maca are boiled and served as juice.

For the present study, the aqueous extract of the hypocotyls was prepared according to the traditional method. In brief, 500 g of the pulverized dried hypocotyls were placed in a container with 1500 ml of water and boiled. The preparation was left standing to cool and was then filtered. Then, the filtrate was lyophilized (Lyophilizer freeze Mobile12).

The freeze-dried aqueous extract of Maca was further diluted. The Maca solutions were placed in small vials and kept in a refrigerator at 4°C until use. Mice received 1 g Maca/kg BW as previously reported [8]. Mice received daily the respective dose according their body weight.

### Treatment

Treatment was administered by oral route. In all experiments an intubation needle N° 18 (Fisher Scientific, Pittsburgh, Pennsylvania) was used to administer 1 g/kg/day of each variety of Maca or vehicle for 21 days. All animal experiments were conducted in compliance with "Guide of the care and use of laboratory animals" as promulgated by the National Research Council [18]. The Institutional Review Board of the Scientific Research Office from the Universidad Peruana Cayetano Heredia approved the study.

### Body and uterine weight

In all experiments, mice were weighted daily during all treatment time. In experiment 1, one day after the last treatment animals were sacrificed by cervical dislocation and uterine were excised and weighed after removal of surrounding adipose tissues. As body weights were not different between control and treated groups, absolute uterine weight will be presented.

### Latent learning: water finding task

The water finding task was performed as described previously [19,20]. Briefly, the apparatus consisted of an open field (30 × 50 × 15 cm) with an alcove (10 × 10 × 10 cm) in the middle of one of the long walls of the enclosure. The inside was painted gray, and the floor of the open field was divided into 15 identical squares with black lines. A drinking tube, identical to that used in the home cage, was inserted into the center of the alcove ceiling with its tip 5 cm (in the training trial) or 7 cm (in the test trial) above the floor. The task consisted of two trials; a training trial (the 1st day) and a test trial (the 2nd day). Both trials were performed from 9:00 am to 3:00 pm.

In the last day of treatment (day 22), the training trial was performed. In this trial, a mouse not deprived of water was placed in one corner of the open field and allowed to freely explore the training apparatus for 3 min. During this time, ambulation was measured by counting the number of times the animals crossed from one score to another in the open field (locomotion count). Also, the frequency of touching, sniffing, or licking of the drinking tube in the alcove (number of approaches) was recorded. Animals that did not begin the exploring within 3 min or did not contact with the drinking tube during exploration were omitted from the test trial. The mouse was immediately returned to the home cage after the training trial and was deprived of water for 24 hr before the test trial. Non-trained mice were prepared for comparison with the trained mice in terms of their ability to find the water source in the same apparatus.

In the test trial (day 22), the trained or non-trained mice were placed in the same corner of the test apparatus. The time lapsed until the mouse entered the alcove was measured as the entering latency. In addition, the time between entering the alcove and drinking the water (finding latency) was also measured. The drinking latency consisted of the sum of the entering and finding latencies. Thus, latent learning was assessed by recording the number of approaches in the training trial and entering, finding and drinking latencies in the test trial.

In this experiment, eighty OVX mice were randomly divided in four groups of 20 OVX animals: a) Control group, OVX mice treated with vehicle; b) OVX mice treated with 1 g/kg of Yellow Maca; c) OVX mice treated with 1 g/kg of Red Maca; d) OVX mice treated with 1 g/kg of Black Maca. Each group was divided in two different groups of ten animals: Non-trained and trained mice.

### Antidepressant activity: forced swimming test

The forced swimming test (FST) is a behavioral test for rodents, which predicts the efficacy of antidepressant treatments [21]. This test induces a immobility state as a reflection of helplessness when subjected to an inescapable situation (tank of deep water). In this paradigm, mice are placed in the tank for a extended period. After an initial swimming period, the mice exhibits an immobility behavior considered a depression-like response. The FST was performed 24-h after the last administration of vehicle or Maca (day 22) from 10:00 am to 2:00 pm.

During the 6 min of the forced swimming test, the duration of immobility was measured as previously described [21]. The apparatus consisted of two Plexiglas cylinders (height: 25 cm, diameter: 10 cm) placed side by side and filled with water (10 cm height) at 23–25°C. Two mice were tested simultaneously for a 6-min period. A non-transparent screen was placed between the two cylinders to prevented the mice from seeing each other. The total duration of immobility, after a delay of 2 min, was measured during a period of 4 min. Each mouse was considered to be immobile when it ceased struggling and remained floating motionless in the water, making only those movements necessary to keep its head above water.

In this experiment, forty OVX mice were divided in four groups (n = 10): a) Control group, b) OVX mice in Yellow Maca (1 g/kg), c) OVX mice in Red Maca (1 g/kg), d) OVX mice in Black Maca (1 g/kg).

### Statistical analyses

Data were analyzed using the statistical package STATA (version 8.0) for personal computers (Stata Corporation, 702 University Drive East, College Station, TX, USA).

Data are presented as mean ± standard error of the mean (SEM). Homogeneity of variances was assessed using a Bartlett test. If variances were homogeneous, differences between groups were assessed by analysis of variance (ANOVA). If the P value in the ANOVA test was significant, the differences between pair of means were assessed by the Scheffé test.

When variances were not homogeneous, the Kruskal-Wallis test was used to assess differences between groups. If the result was statistically significant differences between pair of medians were assessed using the Mann-Whitney-U test.

A value of *P *< 0.05 was considered to be statistically significant.

## Results

### Effect of three different varieties of maca on body and uterine weights

Table 1 shows the body and uterine weight of OVX mice treated during 21 days with three different varieties of Maca. Body weight did not differ between Yellow, Red, Black Maca and control group (P:NS). An increase in uterine weight was observed in OVX mice treated with Yellow, Red and Black Maca with respect to control group (P < 0.05). No differences were observed in wet uterine weight among the three varieties assessed.

**Table 1:** Effect of three different varieties of *Lepidium meyenii *(Maca) on body and uterine weights in OVX mice.

	**Vehicle (n = 20)**	**Yellow Maca (n = 20)**	**Red Maca (n = 20)**	**Black Maca (n = 20)**
**Body weight (g)**	34.21 ± 0.30	35.15 ± 0.35	34.15 ± 0.32	34.73 ± 0.50
**Uterine weight (mg)**	35.51 ± 2.95	69.62 ± 4.73*	60.80 ± 3.39*	72.68 ± 6.14*

Data are mean ± SEM. The number of mice per group is in parenthesis. *P < 0.05 with respect to control group (vehicle).

### Effect of three different varieties of maca on latent learning in the water finding task

In the training session (day 21), no differences were osberved between OVX mice treated treated with Maca and control group (data not shown).

**Figure 1:**
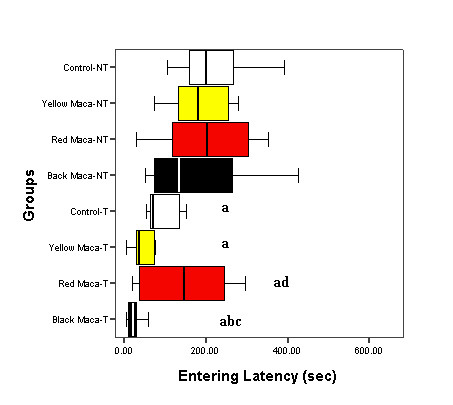
**Effect of Yellow, Red and Black Maca on entering latency in non-trained (NT) and trained (T) OVX mice using the water finding task**. Data are presented as mean ± SEM. ^a^P < 0.05 with respect to non trained control group; ^b^P < 0.05 with respect to trained control group; ^c^P < 0.05 with respect to non trained mice treated with Black Maca; ^d^P < 0.05 with respect to non trained mice treated with Red Maca.

**Figure 2:**
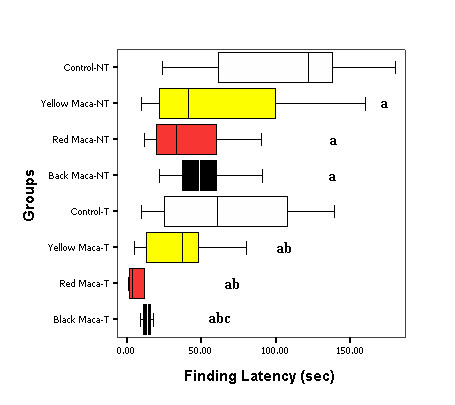
**Effect of Yellow, Red and Black Maca on finding latency in non-trained (NT) and trained (T) OVX mice using the water finding task**. Data are presented as mean ± SEM. ^a^P < 0.05 with respect to non trained control group; ^b^P < 0.05 with respect to trained control group; ^c^P < 0.05 with respect to non trained mice treated with Black Maca.

**Figure 3:**
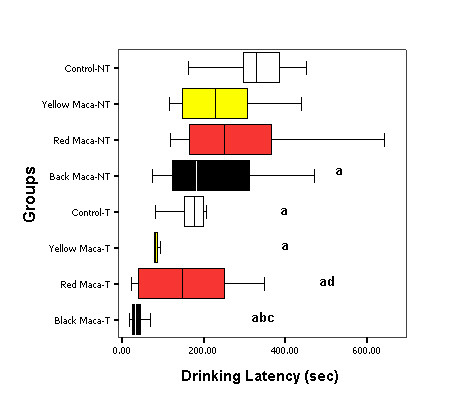
**Effect of Yellow, Red and Black Maca on drinking latency in non-trained (NT) and trained (T) OVX mice using the water findingtask**. Data are presented as mean ± SEM. ^a^P < 0.05 with respect to non trained control group; ^b^P < 0.05 with respect to trained control group; ^c^P < 0.05 with respect to non trained mice treated with Black Maca; ^d^P < 0.05 with respect to non trained mice treated with Red Maca.

Training reduced entering latency (P < 0.05), finding latency (P < 0.05) and drinking latency (P < 0.05) in all groups of treatment (control and Maca treated groups) compared with the non trained mice (Figures 1, 2, 3).

Figure 1 shows entering latency data in non trained (left) and trained (right) mice. Any variety of Maca changed entering latency in non trained mice. However, Black Maca was the only effective to reduce entering latency in trained mice as compared with the trained control group (P < 0.05). In addition, Red and Black Maca reduced entering latency in trained mice with respect to non trained mice treated with these two varieties (P < 0.05). Trained mice treated with Black Maca showed lower values in entering latency than trained mice treated with Red Maca (P < 0.05).

Figure 2 shows finding latency in non trained and trained mice. Yellow, Red and Black Maca reduced finding latency in non trained and trained mice (P < 0.05). Black Maca (P < 0.05) reduced further the finding latency in the trained mice as compared with the non trained mice.

Figure 3 shows drinking latency in non trained and trained mice. Yellow (P < 0.05) and Black Maca (P < 0.05) reduced drinking latency in non-trained group when compared with controls (Figure 3, left side). In trained mice, only Black Maca reduced drinking latency when compared with trained control mice (Figure 3, right side). Moreover, Red and Black Maca reduced drinking latency in trained mice with respect to non trained mice treated with these two varieties (P < 0.05). Trained mice treated with Black Maca showed lower values in drinking latency than trained mice treated with Red Maca (P < 0.05).

### Effect of three different varieties of maca on immobility time using the forced swimming test

**Figure 4:**
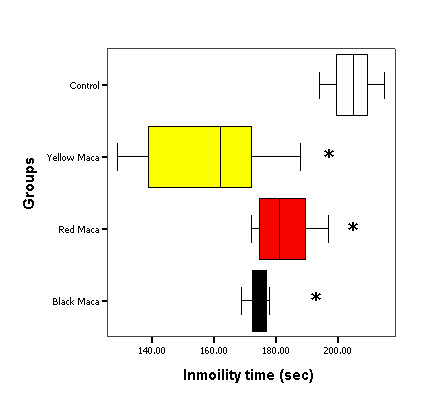
**Immobility time, in the forced swimming test, inOVX mice treatedwith three different varieties of *Lepidium meyenii *(Maca)**. Data are mean ± SEM. *P < 0.05 with respect to control group.

Figure 4 shows the effect of three different varieties of *Lepidium meyenii *(Maca) on immobility time in OVX mice treated during 21 days.

Immobility time was reduced in OVX mice treated with Yellow (157.29 ± 8.32; mean ± SEM), Red (178.86 ± 6.01) and Black (175.29 ± 1.82) Maca with respect to OVX mice treated with vehicle (204.63 ± 2.45; P < 0.05). Yellow Maca had the lowest mean immobility time when compared with the group treated with Black Maca (P < 0.05).

## Discussion

Maca, a traditional food crop from the Peruvian highlands [22], is used for its supposed libido stimulant effect [9-11,16], sperm production [2-7] and its effect on fertility [1,8]. Maca is naturally present in different varieties which are characterized by their external color [12,23]. Recently, different biological effects were described when Yellow, Red and Black Maca were assessed [13,14].

Effects of Maca on mood have also been described in men and rodents [15-17]. After menopause it is possible to find an adverse effect on cognitive function and depressive disorders due to estrogen deficiency [24-27]. Maca has been showed to decrease scores for test to assessment of depression [15,16]. In addition, ovariectomized rats treated with ethanol extract of Maca showed an improve in the bone mass suggesting that is a potentially useful for postmenopausal osteoporosis, which occurs in women as a result of estrogen deficiency [28].

The present study was designed to elucidate the effect of three varieties of *Lepidium meyenii *(Maca): Yellow, Red and Black Maca on learning and depression in OVX mice. We evaluated latent learning using the water finding task according to the method described by Ichihara et al [20]. Mainly, the water finding task assessed memory related to the spatial and attentional construction of the test apparatus and to the specific objects in it [29]. In addition, this task is considered to be a latent learning paradigm and to be related to the ability to sort sensory information and to attention [20,30,31]. In this task mice were not reinforced either positively or negatively by water in training trials. Mice were deprived of water before the test trial in order to promote recall of the location of the water tube in the test apparatus in which they have been exposed in the training trial. The end of the water tube is set further above the floor in the test trial than in training to decrease the probability of it being found by chance [29]. The present study shows the favorable effect of Maca on these tests. Maca had more effects on finding latency and drinking latency. The better effect was observed with Black Maca which it reduced the latency of the three tests (entering, finding and drinking latencies). These outcomes seems not to be related to an increase in motor activity and/or changes in motivational properties to find the water tube, since there are no differences in exploratory behavior and number of approaches to the drinking tube in the training session between groups. It is also possible that an unspecific effect of Maca improving action on physical performances.

Apart from the steroidal estrogens, it has long been known that a large variety of exogenous compounds, including phytoestrogens, mimic the actions of endogenous estrogen to different extents [32]. Major phytoestrogens are flavonoids such as quercetin [33]. Other authors indicate that quercetin has a protective role on learning and memory [34,35]. Previous studies demonstrated that Maca hypocotyls contain flavonoids such as quercetin [36]. Also, the presence of anthocyanins has been reported in Maca hypocotyls [37]. The effects of anthocyanins on nervous systems were reported previously [38-40]. Other authors suggested that neuro-protective effects of estrogens are dependent not on their genomic properties as hormones but rather on their basic chemical properties as hydrophobic phenolic molecules [32]. In fact, phenolic compounds such as 2,4,6-trimethylphenol, N-acetylserotonin, and 5-hydroxyindole exhibit neuro-protective effects without any estrogenicity [32]. We and others have previously demonstrated that estradiol was not affected by treatment with Maca [3,41]. From this, Maca could be acting by another mechanism independent of estrogen activity as previously suggested [8].

In the forced swimming test, we observed that all three varieties, Yellow, Red and Black Maca reduced the immobility time in OVX mice without differences between them. The antidepressant activity of Maca could be due to the presence of phytoestrogens such as quercetin and anthocyanins. Quercetin presents antidepressant activity [42]. However, it is possible that other principles may be acting as anti-depressants, since in previous study it has been demonstrated that scores for depression were reduced after Maca treatment without changes in serum estradiol levels [3,16].

The three varieties of Maca (Yellow, Red and Black) increased uterine weight in OVXmice. This outcome was in accordance with others authors [8]. It is suggested that the effect of Maca increasing uterine weight were not due to an estrogenic effects but to a progestin-like one.

It is still unknown the active secondary metabolites present in the plants responsible for the Maca actions. Some novel compounds have been recently identified, as two new imidazole alkaloids (lepidine A and B) [43]. Also, a benzylated product, named Macaridine, derivative of 1,2-dihydro-*N*-hydroxypyridine, together with the benzylated alkamides (Macamides), *N*-benzyl-5-oxo-6E,8E-octadecadienamide and *N*-benzylhexadecanamide, as well as the acyclic keto acid, 5-oxo-6E,8E-octadecadienoic acid have been described [44]. However, the effect of these compounds has not been assessed for any of the functions described for Maca including learning and depression. One of the limitations of the study is the lack of use of standard reference drug.

## Conclusion

In summary, data presented in this study show that there are differences between the varieties of Maca assessed (Yellow, Red and Black). Black Maca presented the better response with respect to latent learning. Also, Yellow, Red and Black Maca reduced the immobility time in the force swimming test and increased the uterine weight in OVX mice.

## Competing interests

The author(s) declare that they have no competing interests.

## Authors' contributions

JR designed the study, carried out the forced swimming test, performed the statistical analysis and drafted the manuscript. MC and SD performed the ovariectomy procedure and carried out the water finding task. MG prepared the aqueous extracts of the three ecotypes of Maca and was in charge of the treatments. GFG designed the study and drafting the manuscript.

## Pre-publication history

The pre-publication history for this paper can be accessed here:


